# Antibacterial effect of chamomile extract irrigant activated with diode laser against *Enterococcus faecalis*

**DOI:** 10.1007/s10103-026-04984-4

**Published:** 2026-08-15

**Authors:** Elsayed Abdallah Eltayeb, Soha Adel Abdou

**Affiliations:** 1 Department of Medical Applications of Laser, National Institute of Laser Enhanced Sciences, Cairo University., Cairo, Egypt; 2 Dental Department, Research Institute of Ophthalmology, Giza, Egypt

**Keywords:** Antibacterial, Chamomile extract, Diode laser, *Enterococcus faecalis*, Irrigant

## Abstract

The aim of the study was to evaluate the antibacterial effect of chamomile extract irrigant activated with a diode laser 980 nm against *Enterococcus faecalis*. Sixty extracted human single-rooted teeth were chosen. Their roots were mechanically prepared using ProTaper rotary system till size F4 taper 0.06, and their crowns were resected at the cementoenamel junction (CEJ). After sterilization, they were infected for three weeks with *Enterococcus faecalis* (ATCC 29212) obtained from the microbiology laboratory. Based on the kind of final treatment used, they were split into six groups at random. Groups A, B, C, D, E, and F are saline, 5.25% sodium hypochlorite (NaOCl), NaOCl activated by diode laser 980 nm, chamomile extract, chamomile activated by diode laser 980 nm, and NaOCl then chamomile activated by diode laser 980 nm, respectively. Both before and after treatment, the antibacterial efficacy was assessed. The percentage of *Enterococcus faecalis* reduction was 0.2% in group (A), 96.4% in group (B), 98.9% in group (C), 75.3% in group (D), 99.00% in group (E), and 99.2% in group (F). The activation of chamomile irrigant with a diode laser 980 nm is an effective method for significant bacterial reduction of *Enterococcus faecalis* from the root canals.

## Introduction

The goal of root canal treatment is to eliminate microbial infection and stop reinfection in the complex canal system [1]. Even with improvements in endodontic procedures, efficient disinfection is still a major obstacle [2].

In complex canal anatomy, traditional methods like mechanical instrumentation combined with chemical irrigants such as sodium hypochlorite (NaOCl) frequently leave behind debris and microorganisms. Inadequate resolution of these deficiencies may result in chronic infections, post-operative discomfort, and, eventually, therapeutic failure [3].

One common root canal irrigant in endodontic therapy is NaOCl. Its low viscosity makes it simple to introduce into the root canal, and its shelf life is acceptable. It is also readily available and reasonably priced [4]. Nevertheless, it has a number of drawbacks, including tissue irritation, high cell toxicity, dentin collagen denaturation and dissolution, lethality to progenitor cells at the periapex, unpleasant flavor, potential for allergies, and hypochlorite accidents [5].

Therefore, there has been a lot of interest in the endodontic community in finding less invasive and more effective cleaning irrigants and techniques [6, 7]. Successful endodontic results depend heavily on eliminating bacteria and debris from the root canal system [8].

Herbal products are becoming more and more common in dentistry and medicine these days. One well-known medicinal plant in the Asteraceae family is German chamomile. It has a variety of biological properties, such as antibacterial, antifungal, and antiparasitic properties. It has been determined that the essential oil and extracts of chamomile contain over 120 different phytochemical components [9]. It has been demonstrated that chamomile possesses powerful antibacterial properties against both gram-negative and gram-positive bacteria [10]. Additionally, this plant’s extract is used to treat mucositis by efficiently inhibiting infections that affect both the gingiva and the oral mucosa [11].

For successful debridement and sterilization of the canal space, even with the best root canal irrigants, the antimicrobial solutions must be delivered and penetrated into the three-dimensional microstructure. This is often not successfully achieved with the traditional root canal irrigation method, which uses a syringe and needle combination [12]. Even after traditional irrigation and apical enlargement, a small amount of bacteria may still remain in the root canals. The effect of irrigation can be increased by using irrigant activation techniques, which have been proposed to improve irrigant dispersion, flow, and disinfection within the canal system. Lasers have been seen as a promising addition to endodontic treatment to increase the effectiveness of the treatment [13].

Therefore, the aim of the study was to evaluate the antibacterial effect of chamomile extract irrigant activated with a diode laser 980 nm against *Enterococcus faecalis*. The null hypothesis proposed that there was no significant difference in antibacterial effect among tested groups.

## Materials and methods

### Calculation of sample size

The sample size was determined using Abdou and Eltayeb’s [14] study as a guide. This study found that the responses within each group exhibited a normal distribution with a standard deviation of 2.16, indicating that the minimum acceptable sample size was 10 per group. If the probability (power) were 0.8 and the estimated difference were 3. For this test, the Type I error probability was 0.05. Two tailed. The sample size was determined using P.S Power 3.1.6 software.

### Selection and preparation of the samples

A total of sixty extracted human single-rooted teeth with completely grown apices were selected. Teeth with cracks, fractures, root resorption, or immature apices were excluded. The samples were screened by visual inspection and digital periapical radiography before inclusion in the study.

A stone bur in a high-speed motor (NSK, Ti-Max, Ultimate XL, Surgic Pro, Japan) was used to remove the crowns of the teeth. The roots’ length was standardized at 14 +/- 1 mm. One millimeter less than the root apex was determined to be the working length. Using the ProTaper rotary system (Dentsply Maillefer, Ballaigues, Switzerland), all roots were mechanically prepared to size F4 taper 0.06. We utilized 2 ml of 2.5% NaOCl irrigant (Chora X ID, Poland) in between each file. The roots were then irrigated with 5 ml of saline solution. The roots were coated on their external surfaces with varnish, and their apices were sealed with the flowable composite (3 M Oral Care, Filtek™ Supreme Ultra Flowable, Germany). These procedures were performed in the microbiology laboratory at room temperature. After being inserted in an Eppendorf tube, each root was sterilized in a steam autoclave for 20 min at 121 °C. Subsequently, all following procedures were carried out under aseptic conditions within a laminar airflow cabinet.

A bacterial solution of *Enterococcus faecalis* (ATCC 29212) obtained from the microbiology laboratory was made and standardized to 0.5 McFarland in brain heart infusion (BHI) broth (Oxoid Ltd, England). After inoculating each root with 5 ml of infected broth, they were placed in an incubator set at 37 °C for three weeks. Every three days, the broth was replaced.

### Laser used in the study

We used in our study a diode laser 980 nm with continuous wave (CW) mode and 2 watts of power (Lasotronix Smartm PRO. laser, Elektoniczna 2 A, 05–500 Piaseczna; Poland). A 320-µm fiber optic tip was inserted in root canal, 2 mm shorter than the working length and was used in a helical movement inside the root canals from apical to coronal for 20 s. Three cycles of 20 s of radiation have been performed with 5-second rest intervals. Throughout the laser activation procedure, the canal was maintained completely filled with the corresponding irrigating solution while the optical fiber was moved in a helical motion.

### Formation of 50% chamomile extract

300 ml of 96% ethanol was used to saturate 150 g of German chamomile powder at room temperature, which was measured using a digital electronic scale. After 72 h, filter paper was employed to purify the solution. The ethanol was vaporized, and the extract was concentrated using a revolving flask evaporator. The extract was refrigerated at 4 °C until it was needed [15].

### Classification and treatment of roots

Sixty roots were classified into five groups according to final treatment protocol. Each group consisted of ten roots. A 27-gauge standard dental needle was used to deliver the irrigating solution, with the needle placed 1 mm short of the working length.

- Group A: Canals were irrigated by 3 ml of saline for one minute. It acts as a control group.
- Group B: Canals were irrigated by 3 ml of 5.25% NaOCl for one minute.
- Group C: Canals were irrigated by 3 ml of 5.25% NaOCl for one minute and subsequently activated by a diode laser 980 nm for one minute.
- Group D: The root canals were irrigated with 3 ml of chamomile irrigant for one minute.
- Group E: Canals were irrigated with 3 ml of chamomile irrigant for one minute and then activated by a diode laser 980 nm for one minute.
- Group F: Canals were irrigated by 3 ml of 5.25% NaOCl for one minute, then 3 ml of chamomile irrigant for one minute, and then activated by a diode laser 980 nm for one minute.

Following the prior method of treatment, 5 ml of sterile saline was used to irrigate all of the root canals.

### Antibacterial evaluation

Each root canal had two samples taken: one prior to the application of the treatment and the second just after. Size 40 paper points were used to take samples. Each infected paper point was placed solely in Eppendorf tubes with 2 ml of BHI broth then the tubes were agitated for 30 s. After successive dilution to 1/10,000, 15 microns of the contaminated broth were cultivated on bile esculin agar plates, and then the plates were put inside an incubator for two days on 37 °C. Once counting all colonies on agar plates, colony-forming units (CFUs) were determined.

### Statistical analysis

The results were analyzed by Shapiro- Wilk and Kolmogorov- Smirnov tests to provide normality, this produced an insignificant P-value (*P* > 0.05), suggesting that all data were derived from normal data. Accordingly, Tukey’s Post Hoc test was used after the One Way ANOVA test to compare the various groups. Statistical analysis was conducted using SPSS 16 ^®^ (Statistical Package for the Social Sciences), GraphPad Prism, and Microsoft Excel.

## Results

**Fig. 1:**
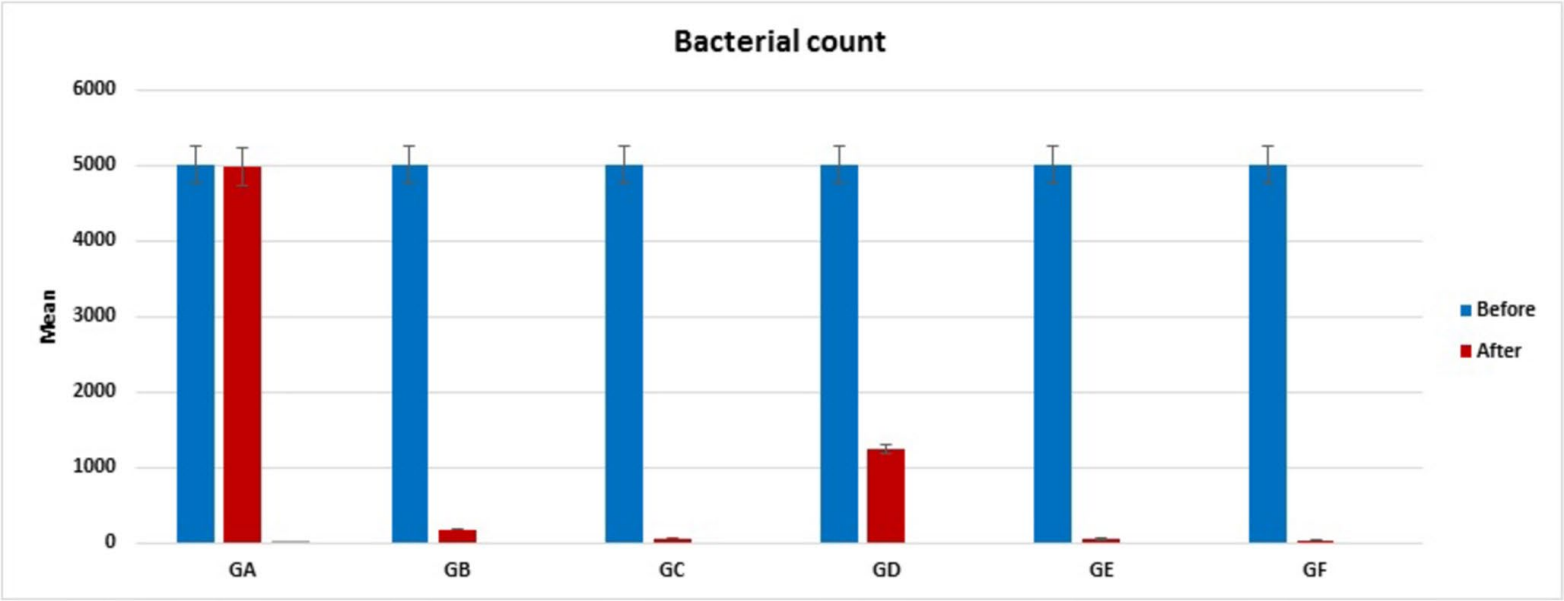
Bar chart showing bacterial count of all groups before and after treatment

**Fig. 2:**
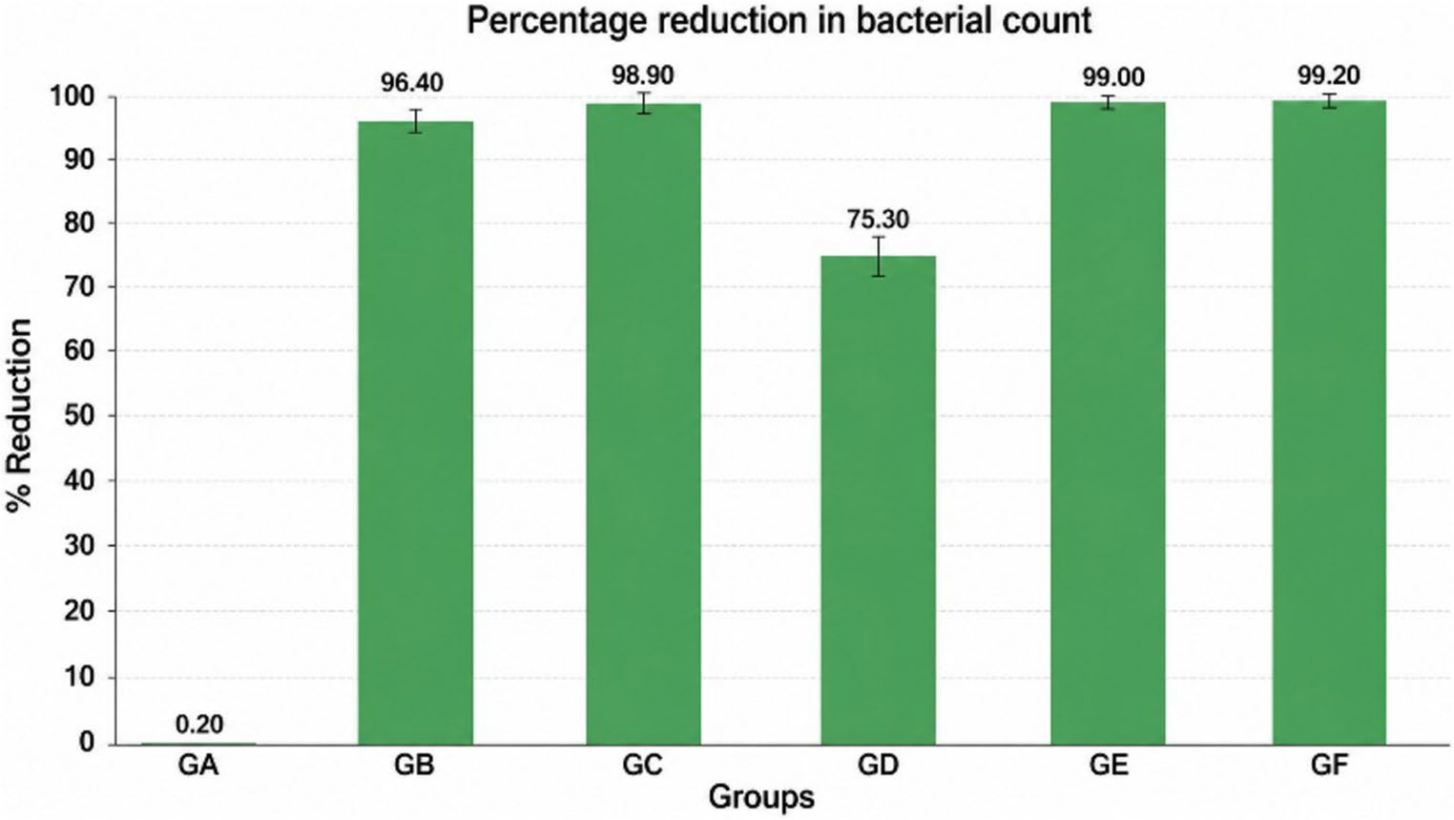
Bar chart showing percentage of change of all groups

Table 1; Figs. 1 and 2 present the bacterial count’s mean and standard deviation for all groups before and after, along with the differences between them and the percentage of change in each group.

**Table 1:** Mean (M) and standard deviation (SD) of bacterial count of all groups

	GA		GB		GC		GD		GE		GF		*P* value
	M	SD	M	SD	M	SD	M	SD	M	SD	M	SD	
Before	5002.90 a	3.75	5001.20 a	4.05	5000.80 a	3.88	5001.50 a	3.57	5002.20 a	5.47	5001.80 a	2.20	0.87
After	4988.00 a	10.62	179.60 b	26.09	57.70 c	9.09	1236.70 d	21.24	48.00 c	5.19	27.00 e	2.00	0.0001*
P value	0.01*		0.0001*		0.0001*		0.0001*		0.0001*		0.0001*		
Difference	−14.90 a	11.20	−4821.50 b	24.40	−4942.90 c	10.22	−3764.60 d	23.64	−4954.20 c	8.47	−4974.80 e	2.94	0.0001*
% of change	−0.20 a	0.42	−96.40 b	0.70	−98.90 c	0.32	−75.30 d	0.67	−99.00 c	0.001	−99.20 c	0.42	0.0001*

* Significant difference as *P* ≤ 0.05

Means with various superscript symbols were significantly different as *P* < 0.05.

Means with similar superscript symbols were insignificantly different as *P* > 0.05

### Comparison between groups (inter-group comparison)

Before treatment: there was an insignificant difference in bacterial amount among groups, as *P* = 0.87.

After treatment: there was a significant disparity in bacterial amount among groups (*P* = 0.0001), as group A (4988.0 ± 10.62) was significantly the highest in bacterial count, while group F (27.00 ± 2.00) was significantly the least in bacterial count.

Differences between before and after treatment: there was a significant disparity in bacterial count among groups (*P* = 0.0001), as group A (14.9 ± 11.2) had significantly the least changes in bacterial count, and group F (4974.8 ± 2.94) had significantly the highest changes in bacterial count.

Percentage of change (percentage of bacterial reduction): there was a significant difference in percentage of bacterial reduction between groups (*P* = 0.0001), as group A (0.2 ± 0.42) had the significantly lowest percentage in bacterial reduction, while group F (99.2 ± 0.42) had the significantly highest percentage in bacterial reduction.

### Comparison between before and after (intra - group comparison)

There was a decrease significantly in bacterial amount in all groups as *P* < 0.05.

## Discussion

The efficacy of root canal therapy correlates with effectiveness of root canal system cleaning and shaping procedure [16]. Mechanical instrumentation is an essential step in root canal disinfection, as it reduces the bacterial load by disrupting the biofilm and removing infected dentin. In our study, ProTaper instrumentation up to F4 was used to standardize canal preparation and provide adequate space for irrigant exchange and diode laser fiber penetration. Therefore, the additional antibacterial effect observed was evaluated after a standardized chemomechanical preparation [17].

*Enterococcus faecalis* was selected for this investigation due to its prevalence in root canals, particularly in reinfection cases, and its increased resistance to endodontic therapy [18, 19].

According to recent systematic reviews, herbal irrigants have demonstrated encouraging antimicrobial activity against endodontic pathogens. However, differences in study design and experimental protocols limit definitive conclusions regarding their routine clinical use [20].

The root canals were exposed to radiation in a helicoidal motion that touched the canal walls, moving from the apical to the coronal region, in accordance with the methods of Godbole et al. [21]. This standardized mechanism ensures that laser light is distributed uniformly inside the root canal and prevents dentin heating, preventing injury to the adjacent periodontal tissues. Garcez et al. [22] have also demonstrated that this procedure enhances the reduction of microbial load.

Evidence from recent reviews suggests that laser-assisted irrigation may enhance the antibacterial effectiveness of conventional irrigating solutions and improve root canal disinfection; however, the strength of the available evidence is still influenced by variations in study methodologies [23].

The findings of our study displayed that the % of reduction in *Enterococcus faecalis* was 75.3% in the group treated with chamomile irrigant (group D). However, when chamomile was activated with a diode laser 980 nm (group E), the percentage of *Enterococcus faecalis* reduction was increased to 99%. Also, the % of reduction in *Enterococcus faecalis* was 96.4% in the group irrigated with 5.25% NaOCl irrigantion (group B). However, when 5.25% NaOCl was activated by a diode laser 980 nm (group C), the % of *Enterococcus faecalis* reduction was increased to 98.9%. This might be explained by the tendency of laser to speed up the irrigating solution’s flow and force it into intricate root canal morphology [24], as laser light penetrates more than 1 mm deep inside the dentinal tubules [25]. Furthermore, the absorption of laser energy by the target tissue results in an increase in temperature. Consequently, the activated irrigating solution induces the breakdown of covalent connections in cellular proteins [26]. Another possibility, as previously reported in Wilson study [27], is that planktonic bacteria are affected by laser light through photochemical changes within live cells. The effect of laser on biofilm may result from the absorption of water in biofilms, and a few remaining live cells may undergo thermal necrosis. These findings were in agreement with the conclusions of Abdou et al. [28], Katalinić et al. [29], and Sardari et al. [30], as they stated that the antibacterial effect of NaOCl irrigant was increased after activation with a diode laser. However, these results were in contrast with the results of Alexander et al. [31], which reported that the antibacterial efficacy of NaOCl irrigantion alone and NaOCl irrigant activated with a diode laser was comparable. This may be attributed to using different parameters of the diode laser. As for chamomile, no research has been conducted yet on using chamomile extract irrigant activated with a diode laser and testing its antibacterial effect.

In the present study, the largest reduction % of *Enterococcus faecalis* was 99.2%, detected in group treated with chamomile irrigant and 5.25% NaOCl irrigantion activated by a diode laser 980 nm (group F). However, this % of *Enterococcus faecalis* reduction was in an insignificant difference with the percentage detected in the group treated with chamomile activated with a diode laser 980 nm (group E), which was 99%, and with the percentage detected in the group treated with 5.25% NaOCl activated by a diode laser 980 nm (group C), that was 98.8%. Chamomile contains several beneficial components, including luteolin, quercetin, apigenin, and chamazolene. These components contribute to chamomile’s medicinal properties, such as antibacterial, antiviral, analgesic, and sedative properties [32]. Chamomile also has the property of being biocompatible with human tissues [15, 33]. These qualities make chamomile an ideal irrigation solution for endodontics [11].

According to Jafari et al. [34], the antibacterial activity of chamomile rapidly increased over time, demonstrating the ability of the material to be adsorbed to dentin hydroxyapatite and released gradually at therapeutic levels. Goes et al. [35] investigated antibacterial efficacy of chamomile, they stated that the chamomile decreased the formation of biofilms in patients with gingival inflammation. Antimicrobial efficacy of chamomilla ethanolic extract has been proven in several experiments [36, 37], with differing outcomes according to the tissue involved [38–40]. According to Shakya et al. [41], chamomilla flowers significantly inhibited the growth of *Enterococcus faecalis*. Also, a study by Mohen et al. [42] stated that chamomile had an inhibitory efficacy against common endodontic microorganisms such as *Candida albicans* and *Enterococcus faecalis*.

The results of the current study proved that the reduction % of *Enterococcus faecalis* in group irrigated by 5.25% NaOCl irrigantion (group B) was more than group irrigated by chamomile irrigant (group D). This may explain the elevated amounts of chlorous acid in NaOCl, which has a significant antibacterial effect by oxidizing microbial enzymes [43]. These results were in agreement with the results of Mohammed and Selivany [15]` and the results of Kameri et al. [44], which stated that a 5.25% NaOCl irrigantion demonstrated superior antimicrobial activity against *Enterococcus faecalis* compared to a chamomile irrigant.

Null hypothesis was rejected due to a large disparity in the percentage of *Enterococcus faecalis* reduction among the examined groups.

In the combined irrigation group, possible chemical interactions between 5.25% NaOCl and chamomile extract should be taken into consideration. NaOCl is a strong oxidizing agent that may react with the organic constituents of herbal extracts, such as flavonoids and phenolic compounds, which could reduce their available active components and affect their antimicrobial performance. Such interactions may also lead to the formation of degradation products or insoluble residues, which might influence the overall cleaning efficacy inside the root canal system [45, 46]. Although no visible precipitation was observed during the experiment, chemical incompatibility between both irrigants cannot be completely excluded and should be further investigated in future studies.

One limitation of our study is the variation in the total volume of the final irrigant among the experimental groups. Group F received a greater total irrigant volume because NaOCl and chamomile extract were applied sequentially before diode laser activation. This difference may have enhanced bacterial reduction through an increased flushing effect in addition to the antimicrobial activity of the tested protocol. Therefore, the antibacterial findings for this group should be interpreted with caution. Future studies should standardize the total irrigant volume among all experimental groups to minimize the influence of this variable.

Another limitation of this study is that temperature changes during diode laser irradiation were not evaluated. The primary objective of the present investigation was to compare the antibacterial efficacy of different irrigation protocols, and assessment of thermal changes was beyond its scope. Although the laser parameters were selected according to previously published studies [47, 48], monitoring intraradicular and external root surface temperature could provide additional information regarding the thermal behavior of the applied irradiation protocol. Future studies are recommended to investigate these temperature changes under the same experimental conditions to further support the safety of diode laser-assisted irrigation.

This study employed a monospecies *Enterococcus faecalis* model that does not fully represent the polymicrobial nature of clinical root canal infections; therefore, it may not completely simulate the in vivo microbial environment. Future studies are recommended to use multispecies biofilm models to better mimic clinical conditions and enhance the translational value of the findings.

Although chamomile extract demonstrated promising antibacterial activity in this study, its preparation involves laboratory-based extraction procedures that may limit immediate clinical applicability. The development of a standardized and commercially available formulation would be necessary to facilitate its practical use in endodontic therapy. Further studies focusing on formulation standardization and stability are required to support its translation into routine clinical practice.

Within the limitations of this in vitro study, diode laser-activated chamomile irrigation showed promising antibacterial potential. Nevertheless, further in vivo and clinical investigations are needed to confirm its effectiveness and safety before clinical implementation.

## Conclusion

The antibacterial effect of chamomile irrigant activated with a diode laser 980 nm against *Enterococcus faecalis* was higher than the antibacterial effect of chamomile irrigant without activation.

## Data Availability

No datasets were generated or analysed during the current study.

## Abbreviations

CEJ: Cementoenamel junction
NaOCl: Sodium hypochlorite
CW: Continuous wave
BHI: Brain heart infusion
CFUs: Colony-forming units
M: Mean
SD: Standard deviation
